# You Do the Math: Coding of Bets and Outcomes in a Gambling Task in the Feedback-Related Negativity and P300 in Healthy Adults

**DOI:** 10.1371/journal.pone.0081262

**Published:** 2013-11-25

**Authors:** Jutta Peterburs, Boris Suchan, Christian Bellebaum

**Affiliations:** 1 Institute of Medical Psychology and Systems Neuroscience, University of Muenster, Muenster, Germany; 2 Institute of Cognitive Neuroscience, Department of Neuropsychology, Faculty of Psychology, Ruhr University Bochum, Bochum, Germany; University of British Columbia, Canada

## Abstract

The feedback-related negativity (FRN) is an event-related potential (ERP) component associated with processing of performance feedback, with more negative amplitudes for losses relative to wins. The amplitude of the FRN following near misses, i.e. the experience of coming close to winning, is between the amplitude elicited by losses and wins. In gambling, however, outcome value may not always be obvious since initially placed bets need to be taken into account when evaluating wins or losses. It is still unclear if initial bet size is reflected in the FRN or the later P300 component. The present study applied a virtual card gambling task to investigate the sensitivity of FRN and P300 to the manipulation of outcome magnitude as implemented through the presence or absence of initial bets, resulting in wins, losses or ambivalent outcomes, with the latter representing losses with and wins without bets. The FRN was larger for trials with bets compared to trials without bets. Wins were associated with a smaller FRN than losses or ambivalent outcomes, while losses and ambivalent outcomes did not differ. P300 amplitudes were larger for trials without bets, and wins were associated with a larger P300 than losses or ambivalent outcomes. Crucially, P300 amplitudes were also smaller for ambivalent outcomes compared to losses. Thus, the different dimensions determining outcome value appear to be integrated in early and late stages of feedback processing. However, only at later stages reflected in the P300 were ambivalent outcomes with and without bet clearly distinguished from other outcomes.

## Introduction

Feedback is essential for informing us about the outcome of our actions and about the success or failure of strategic behaviour and behavioural adaptations. Negative feedback usually signals failure and thus decreases the occurrence rate of preceding actions, while positive feedback commonly indicates success, hence increasing occurrence rate.

Electrophysiological research applying electroencephalography (EEG) has identified an event-related potential (ERP) component specifically associated with feedback-processing, the feedback-related negativity (FRN), a negative deflection with frontocentral distribution occurring approximately 200 to 300 ms after onset of performance feedback [1]–[4]. The FRN has been shown to be larger when outcomes are unfavourable compared to favourable [5]–[7], and is thought to reflect phasic decreases in dopaminergic signals conveyed from the basal ganglia to the anterior cingulate cortex [2], [8]–[10].

It has initially been argued that action outcomes are generally coded in a dichotomous manner in the FRN, i.e. as either signalling goal achievement or failure. For instance, feedback indicating an “even” draw, i.e. neither a win nor loss, has been shown to elicit an FRN comparable to that elicited by negative feedback [11], [12]. Reward magnitude was not reflected in the FRN [12], [13]. More recent studies which took into account subjective reward expectations, however, did report that the FRN reflected gradual deviations of (negative) outcomes from reward expectations, this effect being most pronounced when feedback could actually be used for learning action-outcome contingencies [10], [14]–[16].

In accordance with these findings, the FRN appears to be modulated by subjective outcome value in gambling tasks. The size of the FRN in response to near misses, i.e. the experience of coming close to winning, has been shown to be between full miss (direct loss) and win, although – objectively – the near miss outcome does not differ from a full miss [17]. In contrast, the P300, a positive centroparietal ERP component starting about 300 ms after onset of a visual stimulus, which is commonly associated with processes of decision making [18], attentional allocation [19] and stimulus evaluation [20], appears to be sensitive to objective outcome valence [21]. Indeed, the P300 has been shown to be larger for wins than for losses, while being similar for losses and near misses [10], [17].

It has been argued that near misses are a potent tool in gambling, motivating gamblers to continue playing and to bet more money, thus likely contributing to the development of (pathological) gambling habits [22]–[24]. Another factor promoting the development of pathological gambling habits may be that modern slot machines, for example, usually produce reinforcing sounds and sights even when the amount won is smaller than the initial spin wager. Such “losses disguised as wins” (LDWs) have been shown to elicit similar skin conductance responses (SCRs) as wins, both being larger than the SCRs for losses, indicating that LDWs are associated with arousal levels similar to wins and may thus be subjectively coded as wins [25].

Even in the absence of LDWs, initially placed bets may be neglected in outcome processing. To date it has not been investigated to what extent bets are coded in the process of outcome evaluation as reflected by the FRN and P300. On the one hand, the FRN is capable of coding different increments of subjective outcome values, even if, as in the case of near misses, objective outcomes fall into two categories, wins and losses. On the other hand, a fallacy of coding LDWs as wins, as is suggested by the finding of similar SCRs for LDWs and wins, leads to the expectation that outcomes which differ objectively (i.e. LDWs and wins) are coded in a similar way, without taking the initial bet into account. The present study therefore investigated the coding of different negative and positive outcome magnitudes in the presence and absence of an initial bet in the ERP. Neurologically healthy adult volunteers completed a virtual card gambling task comprising wins and losses of different magnitudes, either with or without an initial bet. Ambivalent outcomes were those which represented a win when no bet was placed and a loss when a bet was required. If the FRN reflects the actual outcome value, both outcome magnitude and the presence or absence of a bet should modulate FRN amplitude. In addition to the FRN, the P300 was analysed to further explore outcome magnitude coding during later, more elaborated stages of outcome processing.

## Methods

### Subjects

Twenty-eight neurologically healthy adult volunteers (15 male, 13 female) were recruited for participation at the Faculty of Psychology of the Ruhr-University Bochum, Germany. Mean age was 24.61 years (SD = 4.76, range 19 to 35 years). All subjects had normal or corrected-to-normal vision and were naïve to the study's intent.

### Ethics

Written informed consent was obtained prior to participation. Subjects received course credits for participation, if applicable. The study conforms to the Declaration of Helsinki and has received ethical clearance by the Ethics Board of the Faculty of Psychology of the Ruhr University Bochum, Germany.

### Procedure

Participants were informed that the experiment involved a computerized card gambling task, and that they would receive a fixed amount of course credits for participation since all bets, wins and losses throughout the task were virtual. After informed consent was obtained, and after demographic information was collected, the electrodes were attached and the experimental task started. Participants were seated in a dimly lit room at a viewing distance of approximately 70 cm from a 19 inch LCD computer screen. The experimental task lasted 60 minutes. Including EEG preparations, the entire test session lasted approximately 90 minutes.

### Experimental task

**Figure 1:**
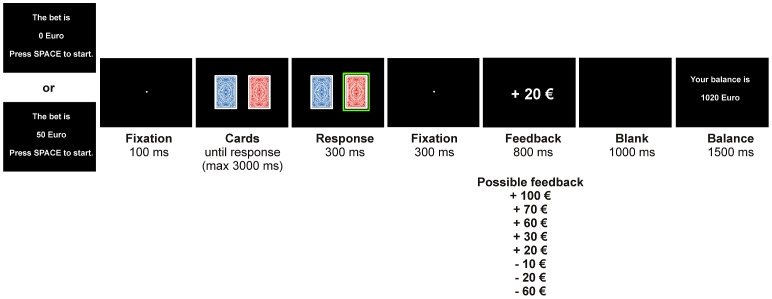
Schematic timeline of stimulus presentation in the gambling task. Participants were informed about the bet at the beginning of each trial, then chose one of two cards by button press before feedback was presented. At the end of each trial, participants were informed about their current account balance.

Participants were instructed to complete a computerized card gambling task with virtual bets, wins and losses, and that they were provided with a starting balance of 1000 €. The sequence of events in the gambling task is illustrated in Figure 1.

At the beginning of each trial, the bet was set to either 0 € or 50 €. Bet size was predetermined on all trials. Participants had to press the space bar to start the trial. After a brief fixation period, two cards were presented for a maximum of 3000 ms. Participants chose one of the cards by button press. Their choice was then highlighted for 300 ms and followed by another fixation period of 300 ms. Subsequent feedback which was presented for 800 ms informed the subject about the outcome of the trial. Possible outcomes were +110 €, +70 €, +60 €, +30 €, +20 €, −10 €, −20 € and −60 €. After an interval of 1000 ms, participants were informed about their current balance at the end of each trial.

Crucially, trial outcomes represented either wins or losses, depending on which bet had been set at the beginning of the trial. While the outcomes +110 €, +70 € and +60 € always represented a win and −10 €, −20 € and −60 € always represented a loss, +30 € and +20 € were ambivalent in that depending on the initial bet they could either be associated with winning or losing. For analysis, trials were therefore averaged according the outcome types “clear win”, “clear loss” and “ambivalent” separately for trials with a bet of 0 € and for trials with a bet of 50 €.

The gambling task comprised 480 trials, with 30 trials per bet and outcome magnitude. The pooled outcome conditions thus comprised 90 trials for wins and for losses per starting bet, respectively, and 60 trials for ambivalent outcomes (for bets of 0 € and 50 €, respectively). Bet and outcome conditions were randomized and balanced throughout the experiment and resulted in a final account balance of 1000 €, thus matching the starting balance. Overall, the mean outcome and thus the expected value was zero. Stimulus presentation was controlled by Presentation software (Neurobehavioral Systems, Albany, California, USA).

### Psychophysiological recordings

EEG was recorded from 30 scalp sites using a Brain Products BrainAmp Standard amplifier (Brain Products, Munich, Germany) and the appropriate software at a sampling rate of 500 Hz. Silver-silver chloride electrodes were fitted to an elastic cap according to the International 10–20 System (F7, F3, Fz, F4, F8, FT7, FC3, FCz, FC4, FT8, T7, C3, Cz, C4, T8, TP7, CP3, CPz, CP4, TP8, P7, P3, Pz, P4, P8, PO7, PO3, POz, PO4, PO8) and referenced to the linked mastoids. Impedances were kept below 5 kΩ.

EEG-data were analyzed off-line using BrainVision Analyzer 2 software (Brain Products, Munich, Germany) and MATLAB (Mathworks, Natick, Massachusetts, USA). Initially, 0.5 Hz high-pass and 40 Hz low-pass filters were applied to the raw data. An independent component analysis (ICA) was then performed on single-subject EEG data [26] in order to correct for blink artefacts. The ICA decomposes the multichannel EEG into a sum of temporally independent and spatially fixed components, the number of components matching the number of channels. Each component can be characterized by a unique temporal and topographical distribution of activation. Components associated with blink artefacts are characterized by a bilaterally symmetric frontal positivity. For each subject, one such component was identified by visual inspection and subsequently removed from the raw data by means of an ICA back transformation. Back-transformed data were visually inspected for a significant reduction of blink artefacts. If the data still contained a number of blink artefacts, a second component was removed.

ERP segments ranging from 200 ms before to 800 ms after feedback onset were created. Baseline correction was performed based on the average signal in the 200 ms directly preceding feedback. Segments containing maximum amplitudes which exceeded an absolute value of 100 µV or a voltage step of 50 µV were excluded by means of automatic artefact detection.

### Statistical analysis

Trials were pooled and averaged according to bet (0 € and 50 €) and outcome type (clear wins, clear losses, ambivalent outcomes). Analyzed ERP components included FRN and P300. Based on visual inspection of the grand-average ERP waveforms, and in following the approach used in a previous study [17], the FRN was defined as mean amplitude in the time window 230 to 330 ms following feedback presentation at electrode FCz. The P300 was defined as mean amplitude in the time window 300 to 550 ms following feedback at electrode Pz. Note that since the FRN refers to a relative negative deflection in the ERP, the term “larger FRN” describes more negative (or less positive) amplitudes. On the other hand, since the P300 refers to a relative positive deflection in the ERP, a larger P300 refers to more positive (or less negative) amplitudes. Repeated-measures analysis of variance (ANOVA) with the within-subjects factors *bet* (0 € and 50 €) and *outcome* (clear win, ambivalent and clear loss) were performed separately for FRN and P300. Greenhouse-Geisser correction was applied if the assumption of sphericity was violated. Post-hoc t-tests were performed to resolve interactions. Thereby, Bonferroni correction was applied to account for multiple testing, setting the corrected significance level to *p*≤.017 one-tailed.

Due to an ongoing debate on whether FRN effects may be better explored by means of a difference signal between negative and positive outcomes [10], [15], additional explorative analysis of the FRN as defined by means of a difference signal, e.g. clear losses – clear wins, were also performed.

## Results

**Figure 2:**
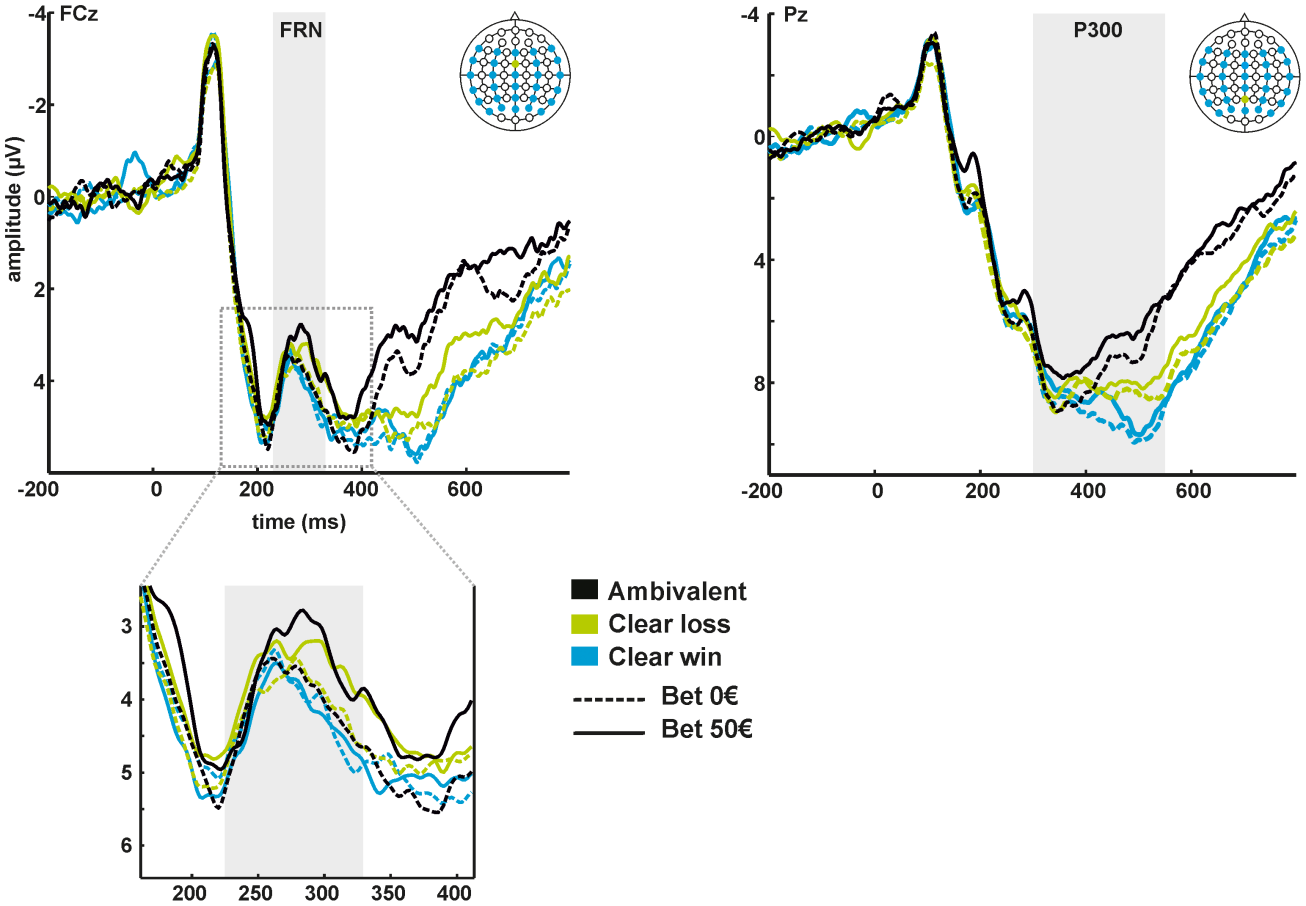
Grand-average ERP waveforms time-locked to feedback onset according to initial bet (0 € or 50 €) and outcome (clear win, ambivalent or clear loss) at electrode FCz (left panel) and Pz (right panel).

**Figure 3:**
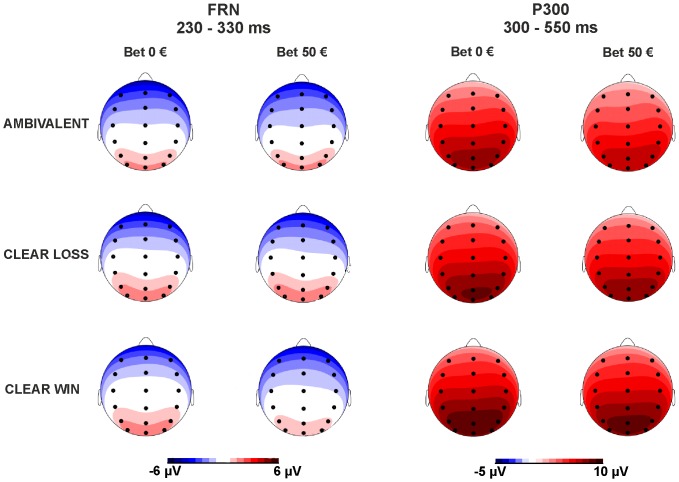
Scalp topographies of FRN and P300 according to initial bet (0 € or 50 €) and outcome (clear win, ambivalent or clear loss). Note that for illustrative reasons, FRN amplitudes are displayed relative to the preceding positive peak, i.e. the P200. For the P300, mean absolute amplitudes are displayed.

Grand-average ERP waveforms according to bet and outcome condition at electrode sites FCz and Pz are provided in Figure 2. Figure 3 depicts scalp topographies of FRN and P300 according to initial bet (0 € or 50 €) and outcome (clear win, ambivalent or clear loss). Note that for illustrative reasons, FRN amplitudes are displayed relative to the preceding P200 peak. For the P300, mean absolute amplitudes are displayed.

### Feedback-related negativity (FRN)

The ANOVA yielded a significant main effect of *bet* (*F*_[1,27]_ = 6.409, *p* = .017), with a larger FRN on trials with bet (3.31 µV±4.35) compared to trials without bet (3.73 µV±4.33). Furthermore, the main effect of *outcome* was significant (*F*_[2,54]_ = 3.247, *p* = .048). Post-hoc tests revealed that the FRN was larger for clear losses (3.37 µV±4.05) compared to clear wins (3.88 µV±4.44; *t*_(27)_ = 2.222, *p* = .017), and for ambivalent outcomes (3.30 µV±4.37) compared to clear wins (*t*_(27)_ = 2.257, *p* = .016). The difference between clear losses and ambivalent outcomes was not significant (*p* = .385). The *bet x outcome* interaction failed to reach statistical significance (*F*_[2,54]_ = 0.775, *p* = .449).

### P300

Analysis of P300 amplitudes yielded a significant main effect of *bet* (*F*_[1,27]_ = 11.709, *p* = .002), with more positive amplitudes, i.e. a larger P300, on trials without bet (7.99 µV±4.47) compared to trials with bet (7.37 µV±4.17). The main effect of *outcome* was also significant (*F*_[2,54]_ = 14.281, *p*<.0001). Post-hoc tests showed that the P300 was larger for clear wins (8.45 µV±4.20) compared to clear losses (7.70 µV±3.99; *t*_(27)_ = 2.338, *p* = .014), for clear wins compared to ambivalent outcomes (6.89 µV±3.99; *t*_(27)_ = 5.043, *p*<.0001), and for clear losses compared to ambivalent outcomes (*t*_(27)_ = −3.397, *p* = .001). Again, the *bet x outcome* interaction did not reach statistical significance (*F*_[2,54]_ = 0.886, *p* = .418).

### Further explorative analyses

In order to further investigate the effect of bet size on FRN and P300, explorative paired-sample *t* tests were performed for net outcomes occurring in both bet conditions, i.e. +60, +20, −20, and −60. For the FRN, a significant difference emerged only for a net outcome of −20 (*t_(27)_* = 2.202, *p* = .036), with a larger FRN for trials with bet (2.61 µV±4.14) compared to without bet (3.63 µV±4.60). The other comparisons failed to reach statistical significance (all *p*>.07), although, descriptively, the FRN was larger for trials with bet in most cases (for net outcomes +60: bet 0 € −4.16 µV±5.32, bet 50 € −4.16 µV±5.26; +20: bet 0 € −3.93 µV±3.93, bet 50 € −3.51 µV±4.17; −60: bet 0 € −3.95 µV±4.05, bet 50 € −3.34 µV±4.34). P300 analyses yielded a similar result pattern: P300 amplitudes differed significantly only for a net outcome of −20 (*t_(27)_* = 2.706, *p* = .012), with a larger P300 for trials without bet (7.76 µV±4.15) compared to trials with bet (6.35 µV±4.18). All other tests failed to reach statistical significance, although descriptively, larger P300 amplitudes were found for the net outcomes +60 (bet 0 €: 9.20 µV±4.79, bet 50 €: 8.6 µV±5.51), and −60 (bet 0 €: 8.29 µV±4.43, bet 50 €: 7.72 µV±4.18), but not for +20 (bet 0 €: 7.45 µV±4.47, bet 50 €: 7.61 µV±4.77), when no bet had been placed.

**Figure 4:**
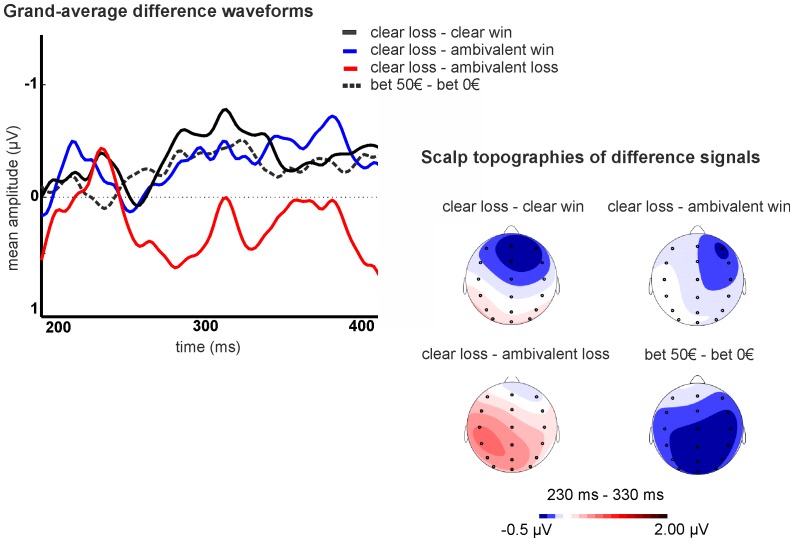
Grand-average difference waveforms and scalp topographies for the difference signals for clear loss – clears win, clear loss – ambivalent win, clear loss – ambivalent loss, and bet 50 € - bet 0 €.

Moreover, since it has been argued that FRN effects may be better explored by means of a difference signal between negative and positive outcomes [10], [15], thereby isolating effects exclusively due to the experimental modulation, grand-average difference signals were calculated for clear losses – clear wins, clear losses – ambivalent wins, clear losses – ambivalent losses, and for bet 50 € - bet 0 €. Grand-average difference waveforms and scalp topographies for mean difference signals in the time window 230−330 ms after feedback are provided in Figure 4. Analysis of the bet 50 € - bet 0 € difference signal showed a negative signal difference with broad centroparietal distribution. Descriptively, the signal difference at FCz was more negative for clear wins – clear losses (−0.51 µV±1.21), followed by clear losses – ambivalent wins (−0.25 µV±1.51) and clear losses – ambivalent losses (0.39 µV±1.40). A somewhat typical FRN topography was only evident for the former two conditions. Paired-sample *t* tests revealed a significantly more negative difference signal (Bonferroni corrected significance level set to *p*<.015) for clear losses – clear wins compared to clear losses – ambivalent losses (*t*_(27)_ = −2.972, *p* = .006), and for clear losses – ambivalent losses compared to clear losses – ambivalent wins (*t*_(27)_ = −3.130, *p* = .004). The comparison between clear losses – clear wins and clear losses – ambivalent wins failed to reach significance (*p* = .303).

## Discussion

The present study aimed to elucidate to what extent initially placed bets are taken into account during feedback processing. More specifically, the study investigated the coding of different negative and positive outcome magnitudes in the presence and absence of an initial bet in the FRN and P300. Twenty-eight participants completed a virtual card gambling task involving three types of trial outcomes: clear wins, clear losses and ambivalent outcomes, the latter representing either a win or a loss depending on whether a bet had been set at the beginning of the trial.

In line with previous findings, the FRN was found to be larger in response to less favourable outcomes [5]–[7]. This was evident in the effect of outcome type: losses were generally associated with a larger FRN than wins. Interestingly, ambivalent outcomes elicited an FRN comparable to losses. At first sight, this finding appears in contrast to recent findings of a graded coding of outcomes by the FRN when feedback probability or magnitude is manipulated [10], , thus supporting previous claims that early feedback processing makes use of dichotomous outcome coding along a good/bad dimension [11]–[12]. Notably however, more fine-grained outcome coding by the FRN has been described in conditions in which feedback could be used for the optimization of behaviour, and when experimental conditions differed clearly with regard to the discrepancy between expected and obtained feedback [10], . While it is likely that, on average, subjects expected an (objective) outcome of 0 € in the present study (corresponding to the mean of all possible outcomes), trial-by-trial reward expectations were not directly assessed.

Importantly, even in the present study, outcomes were not merely coded in a binary fashion in the FRN. The second factor determining outcome magnitude, i.e. the initial bet, was also reflected in FRN amplitudes. Irrespective of outcome type, amplitudes were more negative when the initial bet had been 50 € as opposed to 0 €. While this pattern is in contrast to previous findings [11], it is consistent with the fact that overall, wins were smaller and losses greater when bets had been placed compared to the no bet condition. Thus, the FRN appears to take bets into account, at least to some extent, in coding outcomes in the present gambling task. This conclusion is further supported by the finding that FRN and P300 amplitudes for an identical net outcome (−20 €) differed depending on initial bet, and by the fact that the signal difference between clear losses and wins was significantly more negative than between clear losses and losses on ambivalent trials (i.e. +20 € and +30 € with an initial bet of 50 €) but did not differ from the difference between losses and wins on ambivalent trials (i.e. +20 € and +30 € with no bet). However, in view of the general outcome effect described above, it is conceivable that under specific circumstances outcome coding with regard to a specific dimension may rely on pooling of different outcomes into one category. Pooling of different outcomes according to subjective outcome value, for instance, has previously been reported, with a small compared to a big win being associated with an FRN comparable to a loss [27]. Moreover, general context factors which could be used to group outcome conditions have also been shown to modulate the FRN. For instance, the FRN was observed to be attenuated when participants indicated not to have trusted preceding feedback [28]. It could also be argued that on the present task, trials with a bet of 50 € might have been more salient, and that more attention was allocated to them, also increasing personal relevance and involvement. Enhanced FRN amplitudes have recently been linked to greater negative emotionality [29]. Importantly, participants were informed that payment was fixed and did not depend on performance prior to starting the experiment, so that increased attention or personal involvement on trials requiring bets appears should not have been expected a priori. Nevertheless, explorative analysis of the signal difference between trials with and without bet revealed a negative signal difference lacking the typical focal frontocentral distribution of the FRN but showing a broad, centroparietal topography instead. This pattern could indicate that early bet-related effects might reflect attention-dependent modulation of the P2 rather than bet-dependent modulation of the FRN.

In contrast to the FRN, later stages of feedback processing as indexed in the P300 reflected the full range of potential outcomes, distinguishing between all three outcome types and between both bet conditions. With regard to processing of visual stimuli, the P300 is commonly associated with processes related to stimulus evaluation [20], decision making [18] and attentional allocation [19]. The exact role of the P300 in feedback processing remains a matter of on-going debate. Previous results indicate that the P300 is larger for positive [9], [10], [17], [30] or negative feedback [31], although the absence of valence effects has also been reported [6], [7]. Generally, the P300 appears to be larger for unexpected compared to expected outcomes, and this effect may be particularly pronounced for positive feedback [14], [30]. As outlined above, reward expectations were not assessed in the present study. Nevertheless, it is likely that more favourable outcomes represented unexpected feedback and were therefore associated with more positive P300 amplitudes. In accordance with this notion, the P300 was generally larger for trials without compared to trials with a bet, the actual outcome always being more favourable in the former than the latter trials.

The P300 effects with respect to the different outcome types are more difficult to interpret. While clear wins were associated with a larger P300 than clear losses, thus corroborating previous findings [17], the smallest P300 was found for ambivalent outcomes, for which P300 amplitude differed significantly from both wins and losses. Coding of outcome magnitude in the P300 is thus not linear. Indeed, losses were always associated with worse outcomes than ambivalent feedback. Yet, the P300 clearly distinguished not only between wins and losses and wins and ambivalent outcomes, but also between losses and ambivalent outcomes. This pattern of results strongly suggests that later feedback processing stages serve to integrate objective and subjective outcome valence, thereby likely subserving decisional and evaluative processes. It appears conceivable that these late processing stages are equipped to specifically identify trials on which the initial bet had been larger than the amount eventually won. Along these lines, LDWs may be specifically coded in the P300. Unfortunately, the present experiment did not apply true LDWs, as ambivalent outcomes did not disguise the loss as has previously been described for example in slot machine gambling [25]. Losses as a result of ambivalent outcomes could easily be identified on the basis of the presence or absence of a bet as well as on the current account balance provided at the end of each trial (see Figure 1). Hence, participants were not intentionally tricked. Neither were they specifically instructed to do the math or to identify trials on which the initial bet was larger than the win. Nonetheless, the present results suggest differential neural processing of trials with larger bets relative to the subsequent wins and thus extend the previously reported findings of increased arousal in response to LDWs [25]. Indeed, anecdotal reports of several participants suggest that ambivalent outcomes in the present study may have been perceived as more frustrating or disappointing than direct losses.

The current findings may yield interesting questions for future clinical research. Previous findings of comparably large SCRs in response to LDWs and wins have been obtained in gambling novices [25], as were the present findings of decoding of different outcome magnitudes depending on the presence and absence of a bet in a gambling task. It should be interesting to explore potential differences in habitual and/or pathological gamblers. More research is needed to specifically investigate to what extent processes of early and late feedback processing are sensitive to true LDWs in habitual and/or pathological gamblers.

## Conclusion

Taken together, the present study investigated the sensitivity of FRN and P300 to the manipulation of outcome magnitude which was implemented through the presence or absence of a bet resulting in clear wins, clear losses and ambivalent outcomes in a virtual card gambling task. Results suggest that different dimensions contributing to objective outcome value, namely the outcome per se and the presence or absence of a bet, are integrated to some extent in both early and late stages of feedback processing. However, only at later processing stages reflected in the P300 were different types of outcomes clearly coded separate from one another.
